# Cysteine Attenuates the Impact of Bisphenol A-Induced Oxidative Damage on Growth Performance and Intestinal Function in Piglets

**DOI:** 10.3390/toxics11110902

**Published:** 2023-11-03

**Authors:** Pengxiang Qin, Shangyuan Ma, Changjin Li, Yanjiao Di, Zihao Liu, Huiru Wang, Yang Li, Shuzhen Jiang, Weiren Yang, Ning Jiao

**Affiliations:** Key Laboratory of Efficient Utilization of Non-Grain Feed Resources (Co-Construction by Ministry and Province), Ministry of Agriculture and Rural Affairs, College of Animal Science and Technology, Shandong Agricultural University, Tai’an 271018, China; 2022120521@sdau.edu.cn (P.Q.); 2022110363@sdau.edu.cn (S.M.); 2022120502@sdau.edu.cn (C.L.); 2022120491@sdau.edu.cn (Y.D.); 2021110346@sdau.edu.cn (Z.L.); 2021110350@sdau.edu.cn (H.W.); li_yang@sdau.edu.cn (Y.L.); szjiang@sdau.edu.cn (S.J.); wryang@sdau.edu.cn (W.Y.)

**Keywords:** cysteine, bisphenol A, growth performance, nutrient digestibility, intestinal function, antioxidant

## Abstract

Bisphenol A (BPA), a kind of environmental toxin, widely impacts daily life. Cysteine (Cys) is a nutritionally important amino acid for piglets. However, it remains unclear whether Cys can alleviate BPA-induced oxidative damage in piglets. The aim of the present study was to explore the protective effects of Cys in BPA-challenged piglets. A total of twenty-four piglets were divided into four groups that were further subdivided based on the type of exposure (with or without 0.1% BPA) in a basal or Cys diet for a 28 d feeding trial. The results showed that BPA exposure decreased the piglets’ average daily weight gain by 14.9%, and decreased dry matter, crude protein and ether extract digestibility by 3.3%, 4.5% and 2.3%, respectively; these decreases were attenuated by Cys supplementation. Additionally, Cys supplementation restored BPA-induced decreases in superoxide dismutase (SOD) and glutathione (GSH), and increases in malondialdehyde (MDA) levels, in the serum and jejunum (*p* < 0.05). Moreover, BPA decreased the jejunal mRNA expression of antioxidant genes, which were restored by Cys supplementation (*p* < 0.05). Cys also restored BPA and increased serum D-lactate levels and diamine oxidase (DAO) activity, and BPA decreased jejunal disaccharidase activity (*p* < 0.05). Further investigations in this study showed that the protective effects of Cys were associated with restoring intestinal barrier integrity by improving the jejunal morphology and enhancing the mRNA expression of tight junction proteins (*p* < 0.05). Collectively, the results herein demonstrated that Cys supplementation attenuated the impact of BPA-induced oxidative damage on growth performance, nutrient digestibility and intestinal function.

## 1. Introduction

Bisphenol A (BPA) ubiquitously impacts daily life because it is commonly found in plastic packaging and many consumer products. Humans and animals can be exposed to BPA through its leaching from plastic into food and water [1]. Studies have reported that BPA can lead to damage by causing mitochondrial dysfunction, which leads to reactive oxygen species (ROS) accumulation [2,3]. Oxidative stress, a common and critical event caused by the excessive production of ROS, is one of the main factors that induces many diseases, such as sepsis and enteritis, and even cancers [4]. Weaned piglets suffer oxidative challenges, which are characterized by cell death, destroyed intestinal structure and functions and, ultimately, individual growth retardation [5,6]. Dietary BPA exposure in piglets has been reported to induce oxidative stress by increasing malondialdehyde (MDA) levels and decreasing the activity of copper and zinc superoxide dismutase in the piglet liver [7]. In addition, dietary BPA supplementation in gestating sows aggravated oxidative stress and disturbed newborn and weaning pigs’ intestinal digestion and absorption functions, which were ameliorated by maternal methyl donor supplementation [7,8]. Therefore, it is necessary to explore appropriate strategies to alleviate BPA-induced oxidative challenges without damaging the environment and causing antibiotic resistance.

Cysteine (Cys), as a kind of amino acid and a carrier of a thiol group, plays a pivotal role in protein synthesis and redox maintenance [9]. Dietary cysteine is consumed by 25% of the first-pass metabolism of piglets and is mainly involved in the synthesis of intestinal mucosal epithelial proteins, such as mucin and glutathione (GSH) [10]. However, the effect of GSH on redox homeostasis is mainly achieved through the reduction of the sulfhydryl group provided by cysteine. As Cys that has transformed from methionine is negligible in enterocytes, dietary supplementation is the major source of Cys for the intestinal mucosa [11,12]. Moreover, adequate dietary Cys can improve piglet growth and is an essential amino acid for the intestinal mucosa. In addition, Cys per se can maintain redox homeostasis but may not be necessary for synthesizing GSH [13]. In addition, it has been reported that lipopolysaccharides damaged intestinal integrity and decreased GSH levels by approximately 50%, which can be restored via dietary cysteine supplementation in piglets [14]. Studies also reported that maternal cysteine intake influenced oxidative status by decreasing the serum MDA concentration by approximately 60% [15]. However, the effects of Cys in alleviating BPA-induced oxidative damage have not been underlined.

Therefore, this study was conducted to explore the protective role of dietary Cys supplementation against BPA-induced oxidative damage in improving piglet growth performance and maintaining intestinal mucosa functions, even with adequate dietary methionine. Therefore, this study could provide a novel application for Cys in attenuating oxidative damage.

## 2. Materials and Methods

### 2.1. Animals, Treatments and Management

A total of 24 healthy 35-day-old crossbred castrated piglets (Duroc × Landrace × Yorkshire) with an initial body weight of 14.45 ± 0.43 kg were randomly divided into four groups. There were six replicates per group and one piglet per replicate. Piglets had free access to water and feed throughout a 28 d feed trial. The experimental diets were formulated according to the National Research Council (2012) [16], except for the standardized ileal digestible (SID) methionine (Met) and Cys levels. Cysteine was purchased from Jiakangyuan Technology Development Company Limited (Beijing, China) and BPA from Sigma-Aldrich (Saint Louis, MO, USA). The control group was fed a basal diet with 0.68% SID sulfur-containing amino acid including 0.47% Met and 0.21% Cys, representing a commercial diet, and the BPA group was fed a basal diet supplemented with 0.1% BPA. Meanwhile, the Cys group was fed a Cys diet with 0.36% SID Met and 0.32% SID Cys, and the Cys + BPA group was fed a Cys diet supplemented with 0.1% BPA. The ingredients and nutrient levels of the experimental diets are shown in Table 1. The experimental diets had balanced levels of SID essential amino acids, metabolic energy and electrolytes.

### 2.2. Growth Performance and Sample Collection

Feed intake and body weights of piglets were accurately recorded in replicates throughout the trial period. The growth performance, including average daily weight (ADG), average daily feed intake (ADFI) and feed/gain ratio (F/G), were measured.

At the end of the experiment, all piglets were fasted for 12 h. Blood was collected from the anterior vena cava. Serum was obtained by centrifuging blood at 3000× *g* for 15 min at 4 °C and stored at −20 °C until analysis. After blood sampling, all piglets were humanely sacrificed. The intestine of each piglet was quickly removed, and 2 cm length segments were cut from the middle part of the jejunum, washed with saline solution, and fixed with 4% paraformaldehyde solution for morphological analysis. In addition, the jejunal mucosa was gently scraped. Subsequently, the jejunal tissue and mucosa were collected, snap-frozen in liquid nitrogen, and then, finally stored at −80 °C for further analysis.

### 2.3. Apparent Nutrient Digestibility Analysis

The feces of piglets were collected continuously for three days before the end of the feed trial. The dry matter (DM), crude protein (CP), ether extract (EE) and crude ash (CA) of feed and feces were measured according to the methods of the Association of Official Agricultural Chemists (AOAC) (2012) [17]. Furthermore, the apparent digestibility of DM, CP, EE and CA was determined using the method of acid-insoluble ash (AIA). In addition, the content of AIA in feed and feces was also determined according to the AOAC (2012). The apparent nutrient digestibility was calculated based on the following formula: apparent nutrient digestibility (%) = (1 − (feed AIA content/fecal AIA content) × (fecal nutrient content/feed nutrient content)) × 100.

### 2.4. Serum Biochemical Indexes

Activity of serum enzymes including lactate dehydrogenase (LDH), alkaline phosphatase (ALP), aspartate transaminases (ALT) and aspartate aminotransferase (AST), and levels of metabolites including glucose (GLU), blood urea nitrogen (BUN), total protein (TP), triglycerides (TG), total cholesterol (TC), albumin (ALB), high-density lipoprotein (HDL) and low-density lipoprotein (LDL), were determined using a COBUS MIRA Plus automatic biochemical analyzer (Roche Diagnostic System Inc, Basel, Switzerland) according to a routine operation.

### 2.5. Serum and Jejunal Antioxidant Assessment

The activity of superoxide dismutase (SOD) and diamine oxidase (DAO) in the serum, and the content of D-lactate, glutathione (GSH) and malondialdehyde (MDA) in the serum and jejunum of piglets, were determined via commercial kits (Nanjing Jiancheng Bioengineering Institute, Nanjing, China) following the manufacturer’s instructions using an ultraviolet–visible spectrophotometer.

### 2.6. Jejunal Morphological Observation

The jejunal morphology was examined routinely. Briefly, the fixed jejunal tissues were dehydrated in ethanol and xylene, embedded in paraffin, and then, sliced into 6 μm thickness slices using a microtome device. Subsequently, the slices were stained with hematoxylin and eosin and sealed with neutral resin. The morphology of the jejunum was observed using a Nikon Elipse 80i microscope (Nikon, Tokyo, Japan), and digital pictures were taken using a DP25 digital camera. In addition, villus height (VH) and crypt depth (CD) were measured from eight digital pictures per group and 40 crypts and villi per picture by an experimental researcher blinded to this experimental protocol. Afterwards, the ratio of villus height to crypt depth was calculated.

### 2.7. Quantitative Real-Time PCR (qRT-PCR) Analysis

Total RNA was extracted from the jejunal mucosa using a Trizol RNA extraction reagent as described in a previous study [18]. Following the manufacturer’s instructions, reverse transcription was conducted using the HiScript^®^ III RT SuperMix (Vazyme, Nanjing, China), and cDNA was used as a template in the subsequent reactions. Real-time PCR was performed using the SYBR qPCR Master Mix (Vazyme, Nanjing, China) on a QuantStudioTM 5 Real-Time PCR instrument (Applied Biosystems, Foster City, CA, USA) following the manufacturer’s instructions. All qRT-PCR experiments were repeated three times. *β*-*Actin* was used as the internal control. The relative target gene mRNA expression was calculated using the 2^−ΔΔCt^ method. The primer sequences and product sizes are listed in Table 2.

### 2.8. Jejunal Disaccharidase Activity Analysis

The activities of lactase, sucrose and maltase in the jejunum were determined using commercial kits (Nanjing Jiancheng Institute of Biological Engineering Co., Nanjing, China) according to manufacturer’s instructions. Briefly, the tissues were added to homogenate medium at a ratio of 1:9, and then, grinded at 2500× *g* for 10 min at 4 °C. The supernatants were collected to determine the activity of disaccharidase using an ultraviolet-visible spectrophotometer at 505 nm.

### 2.9. Statistical Analysis

All the data were statistically analyzed via one-way ANOVA using SAS (version 9.4, SAS institute, Cary, NC, USA) followed by the Student–Newman–Keuls test. Results are expressed as means ± SEMs. Differences were considered significant at a *p* value < 0.05. The figures were drawn using GraphPad Prism (version 8, La Jolla, CA, USA).

## 3. Results

### 3.1. Piglet Growth Performance

**Figure 1:**
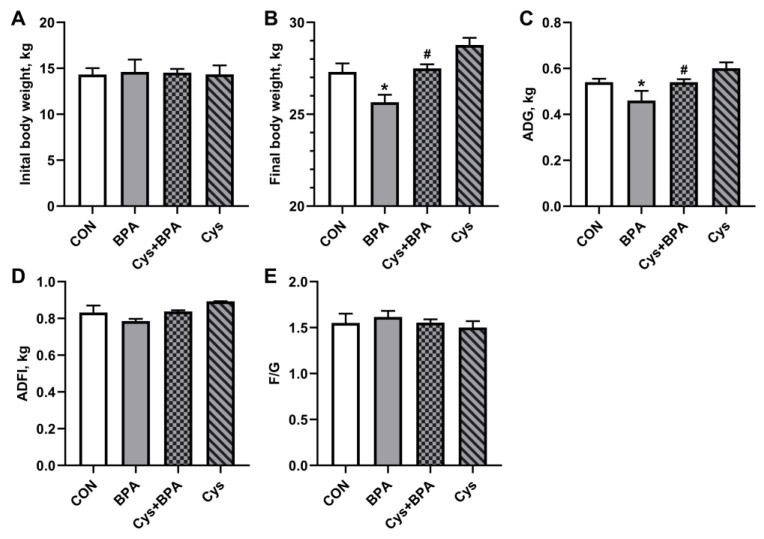
Effects of dietary cysteine supplementation on BPA-challenged piglet growth performance. (**A**) Initial body weight. (**B**) Final body weight. (**C**) Average daily gain (ADG). (**D**) Average daily feed intake (ADFI). (**E**) The ratio of feed to gain (F/G). CON group were fed a basal diet. BPA group were fed a basal diet supplemented with 0.1% BPA. Cys group were fed a Cys diet. CON + BPA group were fed a Cys diet supplemented with 0.1% BPA. Results are expressed as means ± SEMs, *n* = 6. * *p* < 0.05 compared to the CON group. # *p* < 0.05 compared to the BPA group.

As shown in Figure 1, there was no significant difference in initial body weight among treatments. However, compared with the control group, BPA decreased piglets’ final body weight and ADG (*p* < 0.05). Cys supplementation reversed the adverse effects of BPA on body weight and ADG (*p* < 0.05). Nevertheless, BPA and Cys supplementation did not influence ADFI and the F/G (*p* > 0.05).

### 3.2. Apparent Digestibility of Nutrients

**Figure 2:**
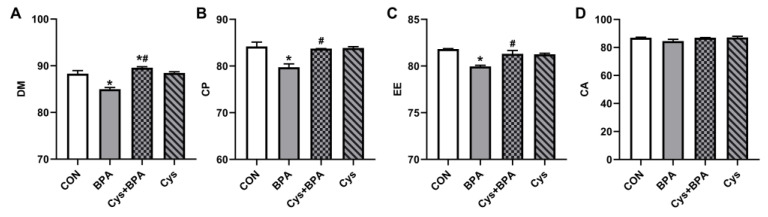
Effects of Cys supplementation on nutrient digestibility in BPA-challenged piglets. (**A**) Dry matter (DM). (**B**) Crude protein (CP). (**C**) Ether extract (EE). (**D**) Crude ash (CA). Results are expressed as means ± SEMs, *n* = 6. * *p* < 0.05 compared to the CON group. # *p* < 0.05 compared to the BPA group.

To investigate the basis of the beneficial effect of Cys on BPA-challenged piglets’ growth performance, the apparent nutrient digestibility of piglets was determined. Figure 2 shows that the DM, CP and EE digestibility of piglets in the BPA group was lower than that of piglets in the CON group (*p* < 0.05). Cys supplementation significantly increased DM, CP and EE digestibility upon BPA exposure (*p* < 0.05) (Figure 2A–C). In addition, DM digestibility was higher in the Cys + BPA group than in the CON group (*p* < 0.05) (Figure 2A). However, BPA exposure and Cys supplementation did not alter the digestibility of CA in piglets (*p* > 0.05).

### 3.3. Serum Biochemistry and Antioxidants

**Figure 3:**
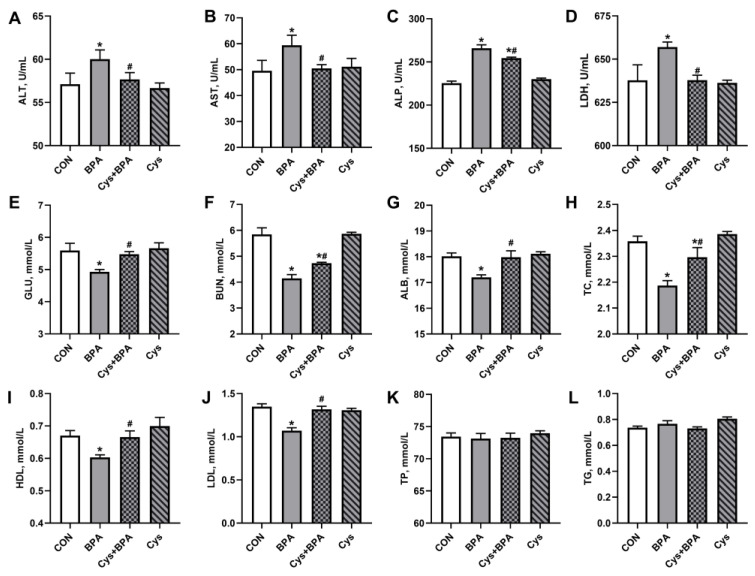
Effects of Cys supplementation on serum biochemistry in BPA-challenged piglets. (**A**–**D**) Serum activity of aspartate transaminase (ALT) (**A**), acrylic aminotransferase (AST) (**B**), alkaline phosphatase (ALP) (**C**) and lactic dehydrogenase (LDH) (**D**), respectively. (**E**–**L**) Serum levels of metabolites including glucose (GLU) (**E**), blood urea nitrogen (BUN) (**F**), albumin (ALB) (**G**), total cholesterol (TC) (**H**), high-density lipoprotein (HDL) (**I**), low-density lipoprotein (LDL) (**J**), total protein (TP) (**K**) and triglycerides (TG) (**L**), respectively. Results are expressed as means ± SEMs, *n* = 6. * *p* < 0.05 compared to the CON group. # *p* < 0.05 compared to the BPA group.

Compared with the control group, the enzyme activity and serum activity of ALT, AST, ALP and LDH in the BPA group was significantly increased (Figure 3A–C) (*p* < 0.05), while the serum levels of metabolites including GLU, BUN, ALB, TC, HDL and LDL were decreased (Figure 3D–I) (*p* < 0.05). However, Cys restored the enzyme activity and metabolite level in BPA-challenged piglets. In addition, compared to control group, Cys increased ALP activity and decreased BUN and TC contents upon BPA exposure (*p* < 0.05). There were no significant differences in the serum levels of TP and TG among the treatments (*p* > 0.05).

**Figure 4:**
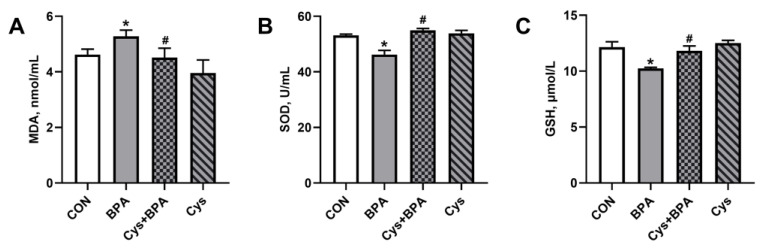
Effects of Cys supplementation on serum antioxidant content in BPA-challenged piglets. (**A**) Malondialdehyde (MDA) content. (**B**) Superoxide dismutase (SOD) activity. (**C**) Glutathione (GSH) content. Results are expressed as means ± SEMs, *n* = 6. * *p* < 0.05 compared to the CON group. # *p* < 0.05 compared to the BPA group.

To assess the attenuated role of Cys upon BPA injury, serum antioxidant content was determined. BPA increased serum MDA content, while it decreased GSH and SOD activity (Figure 4) (*p* < 0.05). Cys attenuated BPA-induced damage by decreasing serum MDA content, and increasing serum SOD activity and GSH content (*p* < 0.05).

### 3.4. Intestinal Morphological Examination

**Figure 5:**
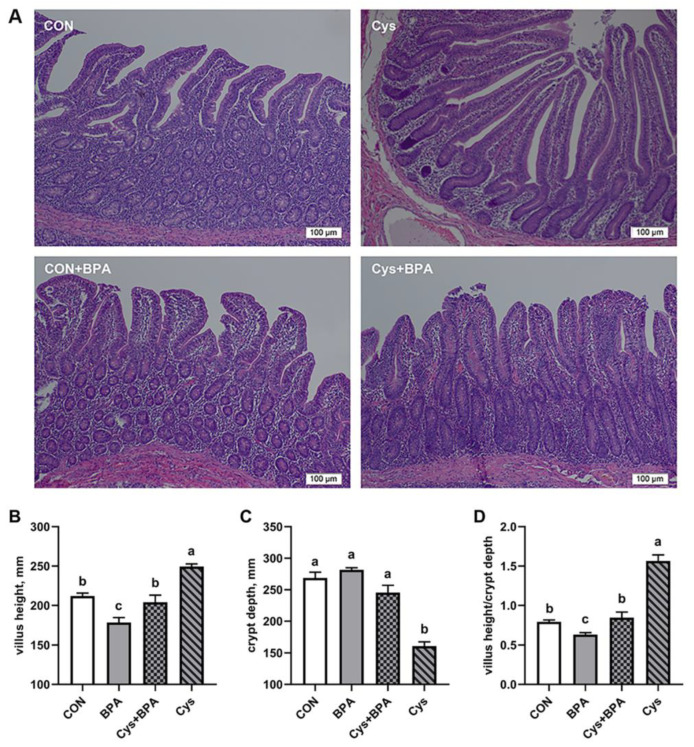
Effects of Cys supplementation on jejunal morphology in BPA-challenged piglets. (**A**) Representative hematoxylin and eosin staining images. (**B**) VH, villus height. (**C**) CD, crypt depth. (**D**) Villus height-to-crypt depth ratio. Results are expressed as means ± SEMs, *n* = 6. Scale bars indicate 100 μm. ^a–c^ Different lowercase letters represent significant differences at *p* < 0.05.

The effects of cysteine on the jejunal morphology in BPA-challenged piglets are shown in Figure 5. Compared with control group, BPA severely broke the jejunal gland epithelium and damaged the jejunal mucosa, which are indicated by shorten villi and a lower ratio of villus height to crypt depth (*p* < 0.05). However, Cys restored jejunal morphological structural damage caused by BPA, which is shown as a basically intact mucosal epithelium, occasional epithelial abscission, higher villi, a higher villus height/crypt depth ratio and a shallower crypt. In addition, dietary supplementation of Cys significantly decreased the crypt depth, but increased the villus height and villus height-to-crypt depth ratio compared to the other group *(p* < 0.05).

### 3.5. Jejunal Antioxidants

**Figure 6:**
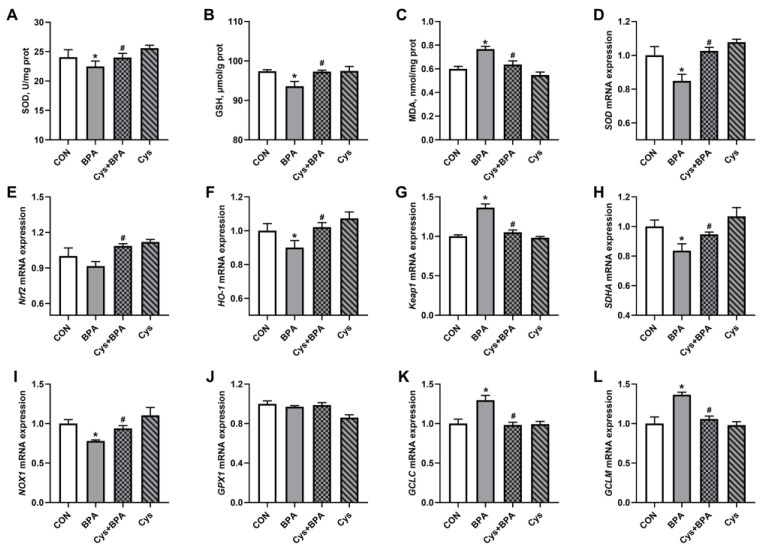
Effects of Cys supplementation on jejunal antioxidant in BPA-challenged piglets. (**A**) Superoxide dismutase (SOD) activity. (**B**) Glutathione (GSH) content. (**C**) Malondialdehyde (MDA) content. (**D**–**L**) mRNA expression of *SOD* (**D**), nuclear factor erythroid-2-related factor (*Nrf2*) (**E**), heme oxygenase 1 (*HO-1*) (**F**), Kelch-like ECH-associated protein 1 *(Keap1)* (**G**), Succinate dehydrogenase subunit A (*SDHA*) (**H**), NADPH oxidase 1 (*NOX1*) (**I**), glutathione peroxidase 1 (*GPX1*) (**J**), glutamate cysteine ligase catalytic subunit (*GCLC*) (**K**) and glutamate cysteine ligase modifier subunit (*GCLM*) (**L**), respectively. Results are expressed as means ± SEMs, *n* = 6. * *p* < 0.05 compared to the CON group. # *p* < 0.05 compared to the BPA group.

As there was an alteration in serum antioxidant content caused by BPA and Cys, jejunal antioxidant content was measured. The SOD activity and GSH content were decreased, while MDA content was increased by BPA exposure in the piglet jejunum (Figure 6A–C) (*p* < 0.05). Cys supplementation could restore SOD activity and GSH and MDA content upon BPA treatment. Furthermore, BPA decreased the mRNA expression of *SOD*, heme oxygenase 1 (HO-1), succinate dehydrogenase subunit A (*SDHA*) and NADPH oxidase 1 (*NOX1*), but increased that of kelch-like ECH-associated protein 1 (*Keap1*), glutamate cysteine ligase catalytic subunit (*GCLC*) and glutamate cysteine ligase modifier subunit (*GCLM*) (Figure 6D–L) (*p* < 0.05). However, Cys attenuated the adverse effects of BPA on antioxidant-related gene mRNA expression (*p* < 0.05).

### 3.6. Intestinal Barrier Function

**Figure 7:**
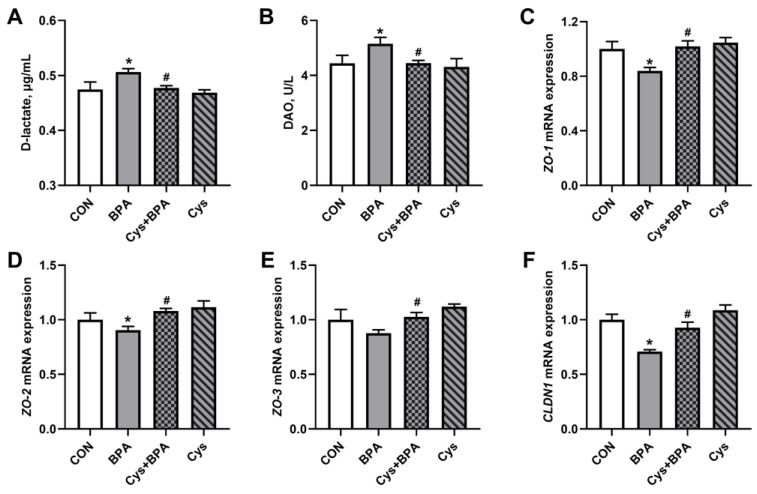
Glutamine alleviated BPA-induced piglet jejunum injury. (**A**) Serum D-lactate content. (**B**) Serum DAO activity. (**C**–**F**) mRNA expression of zona occluden-1 (*ZO-1*) (**C**), zona occluden-2 (*ZO-2*) (**D**), zona occluden-3 (*ZO-3*) (**E**) and claudin-1 (*CLDN1*) (**F**), respectively. Results are expressed as means ± SEMs, *n* = 6. * *p* < 0.05 compared to the CON group. # *p* < 0.05 compared to the BPA group.

To gain an insight into the beneficial effects of Cys on the intestinal barrier function in BPA-challenged piglets, we assessed serum D-lactate content and DAO activity, and the mRNA expression of tight junction proteins. As shown in Figure 7, BPA significantly increased serum D-lactate content and DAO activity, which were decreased by Cys supplementation (*p* < 0.05). In addition, jejunal zona occluden-1 (*ZO-1*), zona occluden-2 (*ZO-2*) and claudin-1 (*CLDN1*) mRNA expressions were significantly increased in the BPA group compared with the control group (*p* < 0.05). Nevertheless, Cys restored the BPA-decreased mRNA expression of *ZO-1*, *ZO-2*, zona occluden-3 (*ZO-3*) and *CLDN1* (*p* < 0.05).

### 3.7. Disaccharidase Activity

**Figure 8:**
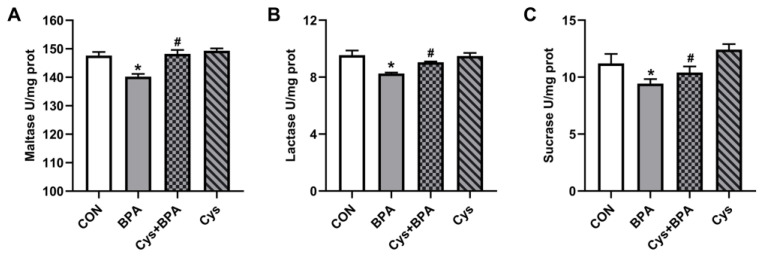
Effects of cysteine supplementation on disaccharidase activity in jejunal mucosa in BPA-challenged piglets. (**A**) Maltase. (**B**) Lactase. (**C**) Sucrase. Results are expressed as means ± SEMs, *n* = 6. * *p* < 0.05 compared to the CON group. # *p* < 0.05 compared to the BPA group.

To examine the protective effects of Cys on intestinal function in BPA-challenged piglets, we determined the disaccharidase activity in the jejunal tissues. According to Figure 8, maltase, lactase and sucrase activity were lower in the BPA group compared with the control group (*p* < 0.05). Cys supplementation restored disaccharidase activity upon BPA challenge (*p* < 0.05).

## 4. Discussion

BPA is a kind of endocrine-disrupting compound in the environment, and can cause oxidative damage to the intestine, liver and reproductive and nervous systems in humans and animals [19,20]. Studies have reported that dietary BPA supplementation in gestating sows could aggravate oxidative stress and impair intestinal morphology, disaccharidase activity and nutrient transporter gene expression in newborn and weaning pigs [7,8]. In addition, it was reported that BPA decreased animal survival and growth performance [2,21]. Consistently, the present study found that BPA significantly impaired growth performance, including final body weight and ADG, which was accompanied by decreased apparent nutrient digestibility, in piglets. Cysteine is a kind of functional amino acid. A study reported that adequate dietary Cys supplementation could optimize piglet growth [2]. Similarly, this study demonstrated that Cys could improve piglet growth performance and nutrient digestibility upon BPA exposure, which indicated that Cys can protect piglets from oxidative damage.

Serum biochemical indexes are important indicators reflecting metabolism and tissue integrity in animals. It was reported that serum AST, ALT, ALP and LDH levels were increased with increasing intestine damage and necrosis [22]. This study demonstrated that BPA exposure significantly increased serum level of AST, ALT, ALP and LDH, which suggests that BPA induced piglet jejunal injury. However, Cys supplementation upon BPA exposure decreased these serum enzyme levels, indicating that Cys could attenuate BPA-induced injury of the jejunum. In addition, BPA altered serum levels of metabolites, which were also restored by Cys supplementation.

Oxidative stress is one of the main factors that induces diseases and cancers, which then leads to piglet growth retardation. The MDA, as a lipid peroxidation product, is associated with oxidative stress-induced damage by altering cell membrane permeability [23,24]. In addition, SOD and GSH, as the major endogenous antioxidants, are the main scavengers of ROS [24,25]. In the present study, BPA increased serum and jejunal MDA content, while it decreased serum and jejunal mucosa SOD activity and mRNA expression, as well as GSH content, in piglets, indicating that BPA induced oxidative damage in piglets. This was consistent with a previous study in mice, which showed that BPA increased jejunal MDA content, and decreased SOD and GSH [2]. However, as a precursor to GSH synthesis for maintaining the thiol state of antioxidants, Cys supplemented in the diet eliminated the oxidation damage induced by BPA, indicated by restored SOD activity and mRNA expression, and MDA and GSH levels, in piglets. This was consistent with a previous study that showed that Cys increased jejunal GSH content [15].

The protection of piglets from damage induced by oxidative stress often involves the antioxidant signaling pathway activity. The *Nrf2-Keap1/HO-1* signaling pathway is a key cellular sensor of oxidative stress whose activation plays a protective role in piglets [26]. *Keap1* is the negative regulatory protein of *Nrf2*. Generally, *Nrf2* activity is maintained at a low level and distributed in the *Nrf2-keap1* complex [27]. Once activated, *Nrf2* dissociates from the complex and induces *HO-1* and *SOD* expression to eliminate ROS [28]. In this study, we found that BPA suppressed *HO-1* mRNA expression and induced *Keap1* expression, which indicates that BPA induced oxidative damage to the jejunum. However, Cys supplementation increased the mRNA expression of *Nrf2* and *HO-1*, while it decreased *Keap1* expression upon BPA exposure. In addition, Cys enhanced jejunal antioxidant capacity by increasing jejunal *SDHA* and *NOX1* mRNA expression. These results indicate that Cys can attenuate BPA-induced oxidative damage. The mRNA expression of *GCLC* and *GCLM* subunits, genes involved in GSH de novo synthesis, were upregulated by BPA exposure, indicating the activation of the adaptive response for survival. Considering that Cys is a major substrate for cellular antioxidant defense, the addition of Cys could conserve GSH to rescue the oxidative stress-induced adaptive response by further decreasing the mRNA levels of genes involved in GSH synthesis.

Nutrient digestibility was positively associated with enterocyte proliferation, which, in turn, affects intestinal morphology and function [29]. In the present study, as Cys supplementation rescued DM, CP and EE digestibility, we then assessed intestinal function upon BPA exposure. The small intestine not only plays an essential role in nutrient absorption, but acts as barrier to defend against harmful substances [30,31]. Villus height, crypt depth and the villus height-to-crypt depth ratio are important indicators used to evaluate small intestinal absorptive capacity [32]. Intestinal villus shortening or loss usually causes nutrient absorption area reduction, malnutrition, diarrhea and a decrease in disease resistance [33]. We demonstrated here that dietary Cys supplementation could improve villus height and the villus height/crypt depth ratio in the jejunum under BPA challenge, indicating that cysteine might be effective in attenuating BPA-induced damage in the intestinal barrier and nutrient absorption. This was similar to a previous study, which reported that low-dose cysteine improved the development of villi [34]. D-lactate is a metabolite produced by intestinal bacteria, and DAO is an intestinal mucosal intracellular enzyme, which is released into the circulation system when the intestinal barrier is compromised [35]. Therefore, serum DAO activity and D-lactate content can serve as markers of mucosal injury and intestinal permeability [36]. This study found BPA exposure increased the serum D-lactate concentration and DAO activity in piglets, which were decreased by dietary Cys supplementation. This is consistent with a previous study that showed that dietary supplementation of 500 mg/kg N-acetylcysteine decreased DAO activity in serum [37]. These results indicated that Cys could restore nutrient digestibility by maintaining intestinal morphology and permeability.

In addition, protecting the integrity of the intestinal mucosal morphology is vital for maintaining normal functions [38]. Tight junction proteins, composed of zonula occludens, proteins occludin and claudin proteins, are the main components of the intestinal epithelial barrier [39], which maintains epithelial permeability and integrity [40,41]. It has been reported that oxidative stress could induce tight junction protein expression downregulation in pig jejunal epithelial cells [42]. In the present study, we found that BPA exposure decreased the expression levels of *ZO-1*, *ZO-2* and *CLDN1*, consistent with a previous study [2]. Moreover, we found that cysteine supplementation could alleviate BPA damage by upregulating the mRNA expression of *ZO-1*, *ZO-2*, *ZO-3* and *CLDN1* in the jejunum of piglets. These results suggest that Cys could improve intestinal barrier function to defend against BPA exposure in piglets.

Moreover, the activities of sucrase, lactase and maltase in the jejunal mucosa can serve as indicators of digestive function and intestinal mucosal maturation [43]. This study demonstrated that BPA significantly decreased sucrase, lactase and maltase activities in the jejunum, which were rescued by Cys supplementation. These results indicate that cysteine alleviated the BPA-induced decrease in intestinal disaccharide activity. Therefore, we speculate that cysteine can attenuate BPA-induced oxidative damage by improving intestinal function.

## 5. Conclusions

In conclusion, BPA exposure exerted oxidative damage that affected piglet growth, nutrient digestibility and intestinal function. Dietary Cys supplementation alleviated BPA-induced damage in piglets by increasing antioxidant capability, maintaining intestinal integrity and improving intestinal function, possibly by activating the Nrf2 signaling pathway, in piglets. Our study provided a novel application for Cys in attenuating oxidative damage.

## Figures and Tables

**Table 1:** Ingredients and nutrient levels of experimental diets (%, as-fed basis).

Items	Control	Cys
Ingredients		
Corn	69.40	69.41
Dehulled soybean meal	5.30	5.30
Extruded soybean	10.00	10.00
Fish meal	3.50	3.50
High-protein dried whey	3.50	3.50
Wheat bran	2.00	2.00
Soybean oil	1.85	1.85
Dicalcium phosphate	0.80	0.80
Limestone	0.40	0.40
Salt	0.25	0.25
L-Lysine·HCl	0.78	0.78
DL-methionine	0.24	0.14
L-tryptophan	0.08	0.08
L-threonine	0.31	0.31
L-cysteine	-	0.11
L-valine	0.21	0.21
L-leucine	0.05	0.05
L-isoleucine	0.14	0.14
L-histidine·HCl	0.18	0.18
L-phenylalanine	0.16	0.16
Alanine	0.20	0.17
Choline chloride	0.08	0.08
Sugar substitute	0.01	0.01
Fragrance	0.05	0.05
Phytase	0.01	0.01
Premix ^1^	0.50	0.50
Total	100.00	100.00
Calculated nutrition levels		
ME (Mcal/kg)	3.35	3.35
Crude protein	16.81	16.81
SID lysine	1.23	1.23
SID methionine	0.47	0.36
SID cysteine	0.21	0.32
SID tryptophan	0.20	0.20
SID threonine	0.73	0.73
Calcium	0.60	0.60
STTD phosphorus	0.44	0.44

^1^ Premix was provided per kg of diet: VA 1750 IU; VD_3_ 200 IU; VE 11 IU; VK_3_ 0.5 mg; VB_1_ 1 mg; VB_2_ 3 mg; VB_5_ 9 mg; pyridoxine 3.0 mg; Biotin 0.05 mg; VB_9_ 0.3 mg; VB_12_ 15 μg; Mn 3 mg; Fe 150 mg; Zn 80 mg; Cu 5 mg; I 0.14 mg; Se 0.25 mg.

**Table 2:** Primer sequences and product sizes for quantitative real-time PCR.

Gene	Primer Sequence (5′ to 3′)	Accession No.	Product Size (bp)
*CLDN1*	F: GGCATCCTGCTGGGACTAAT R: GCAACTAAGATAGCCAGACCTGAA	NM_001244539.1	149
*GCLC*	F: CGGTGGAGGACAATATGAGGAA R: AGCCTAATCTGGGAAATGAAGTGA	XM_003482164.4	99
*GCLM*	F: GGACAAAACCCAGTTGGAGC R: CAGTTAAATCGGGCGGCATC	XM_001926378.4	104
*GPX1*	F: GCAACCAGTTTGGACATCAGGAA R: CGAAGAGCGGGTGAGATTTG	NM_214201.1	147
*HO-1*	F: AGGCTGAGAATGCCGAGTTC R: TGTGGTACAAGGACGCCATC	NM_001004027.1	90
*Keap1*	F: GTGTTACTACCCAGAGAGGAATGA R: CCGCAGCATAGATACAGTTGTG	NM_001114671.1	104
*NOX1*	F: GCATCCCTTTACCCTGACCT R: CTCAATCCTTGGAACTGGCG	XM_021079610.1	130
*Nrf2*	F: TCAGCACCTTGTACCTTGAAGT R: TTGCCATCTCTTGTTTGCTGC	XM_021075133.	100
*SDHA*	F: ATGGAAAACGGGGAGTGTCG R: TTCCGGTAGCGACAACAGTG	XM_021076930.1	97
*SOD*	F: AAGGGGAATTGCTGGAAGCCA R: CGACGGATACAGCGGTCAACT	NM_214127.2	81
*ZO-1*	F: GCATGATGATCGTCTGTCCTACC R: CCGCCTTCTGTATCTGTGTCTTC	XM_013993251.1	108
*ZO-2*	F: GCAGAGACAACCCCCACTTT R: CGTTAACCATGACCACCCGA	NM_001206404.1	117
*ZO-3*	F: CTGTGGTTGTGTCTGACGTGGTAC R: GGCTATCTTGACGCAGGTCTTGAG	XM_005661351.3	120
*β-Actin*	F: CCACGAAACTACCTTCAACTC R: TGATCTCCTTCTGCATCCTGT	NM_001170517.2	131

## Data Availability

The datasets used and analyzed during the current study are available from the corresponding author upon request.
